# Healthcare providers' perspectives on the Canadian Caries Risk Assessment Tool implementation in Indigenous pediatric primary care: a qualitative study

**DOI:** 10.1186/s12903-025-06036-9

**Published:** 2025-05-10

**Authors:** Olubukola O. Olatosi, Robert J. Schroth, Daniella DeMaré, Betty-Anne Mittermuller, Maria Manigque, Jeanette Edwards, Maryam S. Amin, Alexandra Nicolae, Josée Lavoie, Julianne Sanguins, Prashen Chelikani, Peter D. Wong, Jesse Lamoureux, Mary Bertone, Katherine Yerex, Rhonda Campbell, The Working Together for early childhood oral health study team

**Affiliations:** 1 Department of Preventive Dental Science, Dr. Gerald Niznick College of Dentistry, Rady Faculty of Health Sciences, University of Manitoba, Winnipeg, Manitoba Canada; 2 Department of Oral Biology, Dr. Gerald Niznick College of Dentistry, Rady Faculty of Health Sciences, University of Manitoba, Winnipeg, Manitoba Canada; 3 Children’s Hospital Research Institute of Manitoba, Winnipeg, Manitoba Canada; 4 Department of Pediatrics and Child Health, Max Rady College of Medicine, Rady Faculty of Health Sciences, University of Manitoba, Winnipeg, Manitoba Canada; 5 Shared Health, Winnipeg, Manitoba Canada; 6 School of Dentistry, University of Alberta, Edmonton, Alberta Canada; 7 Toronto Public Health, Toronto, Ontario Canada; 8 Ongomiizwin Research – Indigenous Institute of Health and Healing, Rady Faculty of Health Sciences, University of Manitoba, Winnipeg, Manitoba Canada; 9 Department of Community Health Sciences, Max Rady College of Medicine, Rady Faculty of Health Sciences, University of Manitoba, Winnipeg, Manitoba Canada; 10 Department of Pediatrics, University of Toronto, Toronto, Ontario Canada; 11 Interlake-Eastern Regional Health Authority, Pine Falls, Manitoba Canada; 12 School of Dental Hygiene, Dr. Gerald Niznick College of Dentistry, Rady Faculty of Health Sciences, University of Manitoba, Winnipeg, Manitoba Canada; 13 College of Nursing, Rady Faculty of Health Sciences, University of Manitoba, Winnipeg, Manitoba Canada

**Keywords:** Caries risk assessment, Barriers, Early childhood, Primary care providers, Indigenous

## Abstract

**Background:**

Early childhood caries remains a significant public health concern among Indigenous children in Canada. Integrating caries risk assessment (CRA) into primary care settings could improve early detection and intervention.

**Objectives:**

This qualitative study explored the barriers to implementing and integrating the Canadian CRA tool into the primary care of First Nations and Métis children in Manitoba, based on the perspectives of non-dental primary care providers (NDPCPs).

**Methods:**

Fifty NDPCPs providing care to Indigenous children under six years of age were purposefully selected from ten Indigenous communities in Manitoba, including Winnipeg, Selkirk, St. Laurent, Swan River, Pine Creek, Camperville, and Pine Falls. The study employed an exploratory design, with data collected through eight focus groups and twelve in-depth key informant interviews conducted between April 2023 and September 2024. All interviews were audio-recorded, transcribed verbatim, and analyzed using inductive thematic analysis with NVivo software.

**Results:**

Participants included pediatricians, family physicians, public health nurses, nurse practitioners, physician assistants, dietitians, and child development workers, with an average age of 41 years (range: 24–61) and 13 years of practice experience (range: 1–40). Thematic analysis identified four major barriers to integrating the CRA tool: (1) Service provider level – time constraint, scope of practice, documentation/referral pathways, and funding; (2) Community level – oral care not priority, separation of dental and general health, lack of transportation, and healthcare distrust; (3) Caregiver and child level – lack of dental insurance, parental willingness, substituting CRA for dental visit; and (4) Provider training and skills – lack of training on fluoride varnish application and dental screening. Despite these challenges, participants expressed a positive attitude toward receiving training on early childhood caries prevention, fluoride application, and the use of the Canadian CRA tool.

**Conclusion:**

This study highlights that NDPCPs in Manitoba recognize the Canadian CRA tool as a valuable resource for improving access to early preventive dental care for First Nations and Métis children. The identified barriers provide critical insights for dental, medical, and allied healthcare providers, offering a foundation for developing strategies, guidelines, and policies to enhance preventive oral health services for Indigenous children in Manitoba.

**Supplementary Information:**

The online version contains supplementary material available at 10.1186/s12903-025-06036-9.

## Introduction

Oral health is an essential component of overall health, yet dental caries remains one of the most prevalent chronic conditions among children globally [1]. Early childhood caries (ECC) is caries experienced in the primary dentition of children under six years of age affecting 1.76 billion children worldwide [2]. In Canada, Indigenous children (First Nations, Inuit, and Métis) experience disproportionately higher rates of ECC compared to their non-Indigenous peers [3, 4]. This disparity is influenced by a complex interplay of social determinants of health, including food insecurity, limited access to dental care, historical trauma, and cultural factors [5]. Addressing these inequities requires integrating preventive oral health measures into primary care settings, where Indigenous children are more likely to receive care [6]. Caries-risk assessment (CRA) is a crucial component of pediatric dental care. It involves identifying and analyzing factors associated with the development of dental caries and developing patient-centered preventive and therapeutic care to reduce the risk of caries [2, 7]. In December 2019, the Public Health Agency of Canada sponsored the development of a novel Canadian CRA tool for preschoolers. This CRA tool was designed based on a systematic review of the literature and Canadian evidence on risk factors to identify children at high risk for dental caries, enabling early interventions such as fluoride varnish application, dietary counseling, and timely referrals to dental care providers [8].

Non-dental primary care providers (NDPCPs), including family physicians, pediatricians, nurse practitioners, and public health nurses, are ideally suited to deliver early preventive oral health care to children, as they typically see children for approximately seven well-child visits by the age of one [9, 10]. By embedding this tool into primary care workflows, healthcare providers can play a critical role in mitigating the burden of ECC in underserved populations, including First Nations and Métis children [11]. NDPCPs are encouraged to deliver preventive oral healthcare for infants and toddlers [12]. However, systemic challenges such as limited resources, insufficient cultural appropriateness, and gaps in provider training often impede the integration of oral health into primary care practices [13, 14].

This study aims to explore the perspectives of NDPCPs on the challenges and barriers to implementing and integrating the Canadian CRA tool in the primary care of Indigenous children in Manitoba. By understanding these perspectives, this research seeks to identify actionable insights that can guide the development of strategies, policies, and training programs to improve oral health outcomes among Indigenous children. This is particularly critical in Manitoba, where Indigenous communities face significant health disparities and where systemic and culturally informed interventions are urgently needed.

## Methods

### Study design

**Fig. 1:**
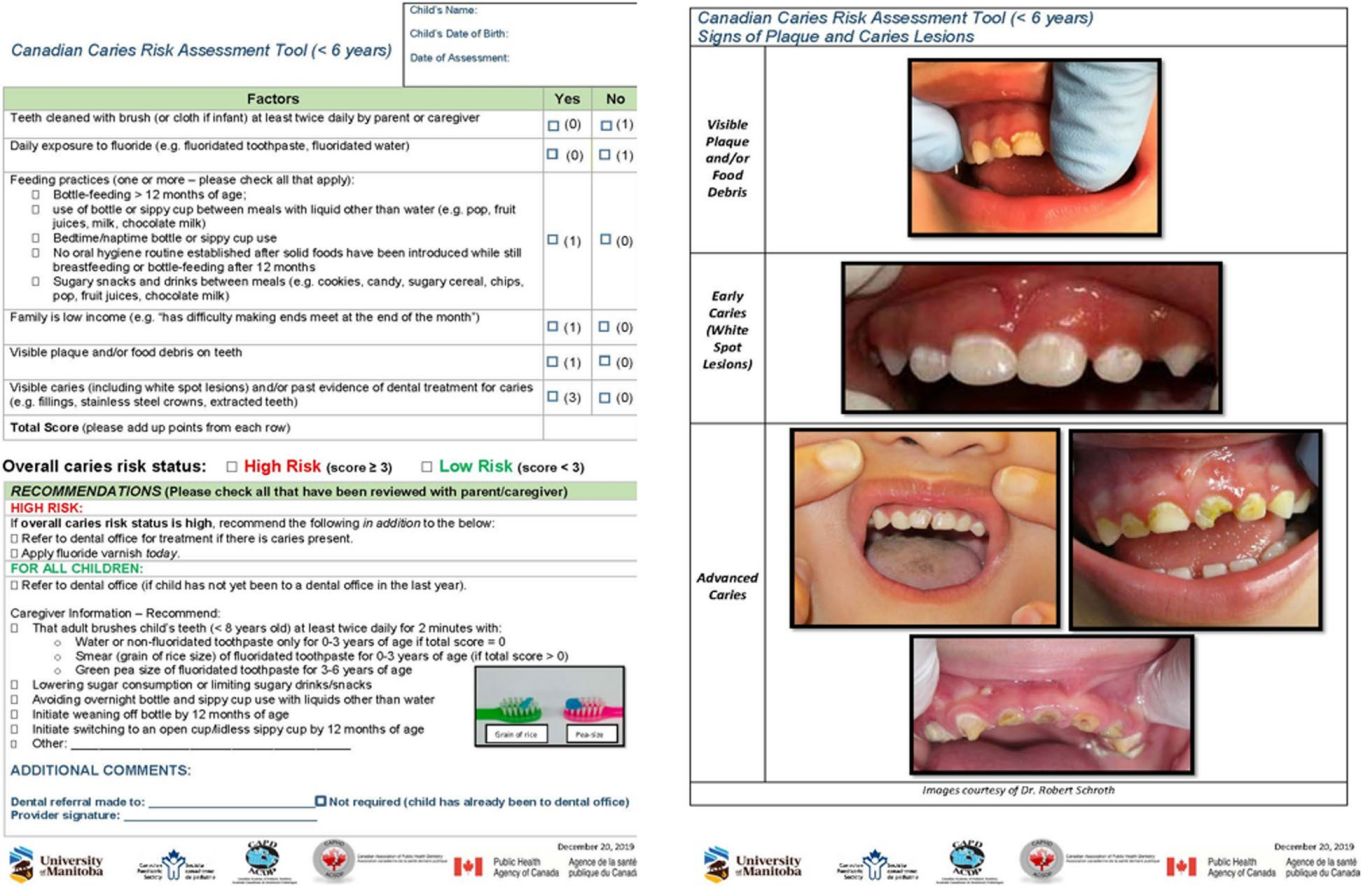
Canadian caries risk assessment tool (< 6 years)

This exploratory qualitative study used semi-structured interviews to explore the perspectives of NDPCPs in Manitoba on the challenges and barriers to implementing and integrating the Canadian CRA tool (Fig. 1) into the primary care for Indigenous children. Guided by a social constructivist research paradigm, the study employed purposeful sampling to ensure critical representation. To accurately interpret participants' responses, researchers utilized a constant comparative method of analysis, enabling the development of authentic conceptual descriptions.

### Ethics

Ethics approval for this study was granted by the University of Manitoba Health Research Ethics Board (HREB) under approval numbers HS25866 (H2023:050). This approval is associated with a University of Manitoba study funded by the Canadian Institutes of Health Research, partnership with First Nations Health and Social Secretariat of Manitoba (FNHSSM) and Manitoba Métis Federation (MMF); ethics approvals HS24621 (H2021:043) and HS20926.

### Research team and reflexivity

Given the qualitative nature of the study, it was essential to emphasize the researchers’ identity, subjectivity, marginality, and perspectives as part of reflexivity and transparency in the research process [15]. The research team was led by RJS, a dental public health specialist and researcher with extensive experience working with First Nations and Métis communities in Manitoba. The primary researcher and first author, OOO, a non-Indigenous Black female pediatric dentist conducted the interviews. Her lived experiences navigating gender, racial and oral health disparities have deepened her understanding of systemic inequities and their impact on health outcomes, particularly for marginalized populations. This perspective informed her research approach to fostering inclusive, community-driven solutions. The research team also included three Indigenous researchers, whose insider positionality facilitated trust-building and access to communities while ensuring that the study was conducted in a culturally respectful manner. To mitigate potential biases in interpreting participants’ responses, several steps were taken. These included ongoing team meetings to critically reflect on positionality, seeking guidance from Indigenous Knowledge Holders, and incorporating feedback from participants in the research process. By integrating diverse perspectives within the team and actively engaging in reflexive practices, we aimed to uphold cultural integrity and minimize interpretive bias in our findings.

### Participants and recruitment

Fifty NDPCPs providing care to First Nations (FN) and Métis children under six years old were purposefully selected from ten health and community centers serving Indigenous communities in Manitoba. These communities included both urban (Winnipeg, Selkirk) and rural/remote locations such as Pine Falls, Swan River, St. Laurent, Pine Creek, Camperville, Thompson, St. Theresa Point, and Berens River. We used purposeful sampling to assure the attainment of critical representation of experiences and ideas by seeking maximum variation of the study participants to include nurses, physicians, physician assistants, dieticians and social workers. Eligibility criteria included being a NDPCP whose client population included Indigenous children aged 6 years below and worked in Indigenous community. NDPCPs were invited to participate through designated contact persons (clinic administrators/managers) at each center, who assisted in disseminating study details. Additionally, recruitment fliers with QR codes linking to detailed information about the study were posted in selected health centers after obtaining the necessary approvals. Participants were also recruited from the University of Manitoba’s Ongomiizwin Health Services and the Department of Pediatrics and Child Health member listings. Prospective participants were provided with information about the study’s objectives, and informed consent forms were emailed to them in advance. These forms were signed and collected on the day of the interview.

### Data collection

A semi-structured interview guide was developed by the study team based on literature reviews and insights from previous investigations [8, 11, 16]. The guide was refined to include additional questions and probes, with the final version reviewed by an interdisciplinary team of experienced researchers specializing in early childhood oral health, health promotion, community development, and Indigenous health (supplementary file). Data were collected through eight focus groups and twelve in-depth key informant interviews conducted between April 2023 and September 2024.

Each focus group included five to eight NDPCPs experienced in caring for Indigenous children. At the start of each session, informed consent and demographic information were obtained. Focus group sessions, lasting 45 to 75 min, were held at community health centers and audio-recorded by OOO, with MM and DD taking field notes. These notes included observations of nonverbal responses and facial expressions to capture participants' emotions during the discussions.

Key informant interviews lasted 15 to 30 min and were designed to delve deeper into individual perspectives. The interview guide was adjusted during the data collection process to incorporate emerging themes, allowing for a deeper exploration of new ideas. All interviews were transcribed verbatim and analyzed using an inductive thematic approach with NVivo© software. Data collection continued until saturation was reached setting the sample size of participants included in the study. To ensure the study’s validity, member checking, expert reviews, field notes, and memos were utilized throughout the process.

### Data analysis

Data analysis was conducted concurrently with data collection, utilizing both inductive and deductive thematic approaches. Transcripts were read and re-read line by line, applying a constant comparative method to deepen the understanding of emerging ideas and concepts. An open coding strategy was used for the initial analysis, with text analyzed line by line and labeled with descriptive words or short phrases that encapsulated the core meaning of the content [17]. Themes were developed by grouping related codes into subthemes and primary themes. Data collection continued until no new information emerged. Initially, we conducted five focus groups and nine key informant interviews, followed by an additional three focus groups and three key informant interviews. Data saturation was determined when no new codes or emerging themes relevant to the study concept were identified in three consecutive interviews. Code frequency counts were used to assess and confirm saturation [18]. To ensure rigor, OOO and another member of the research team independently analyzed the data, then convened to compare codes, themes, subthemes, and their relationships, ensuring inter-coder reliability. The analysis was iterative, with the data and codes revisited and refined as new insights emerged. Results and interpretations were also shared with field experts for validation, and member checking was conducted by providing participants with fact sheets to gather their feedback.

## Results

**Fig. 2:**
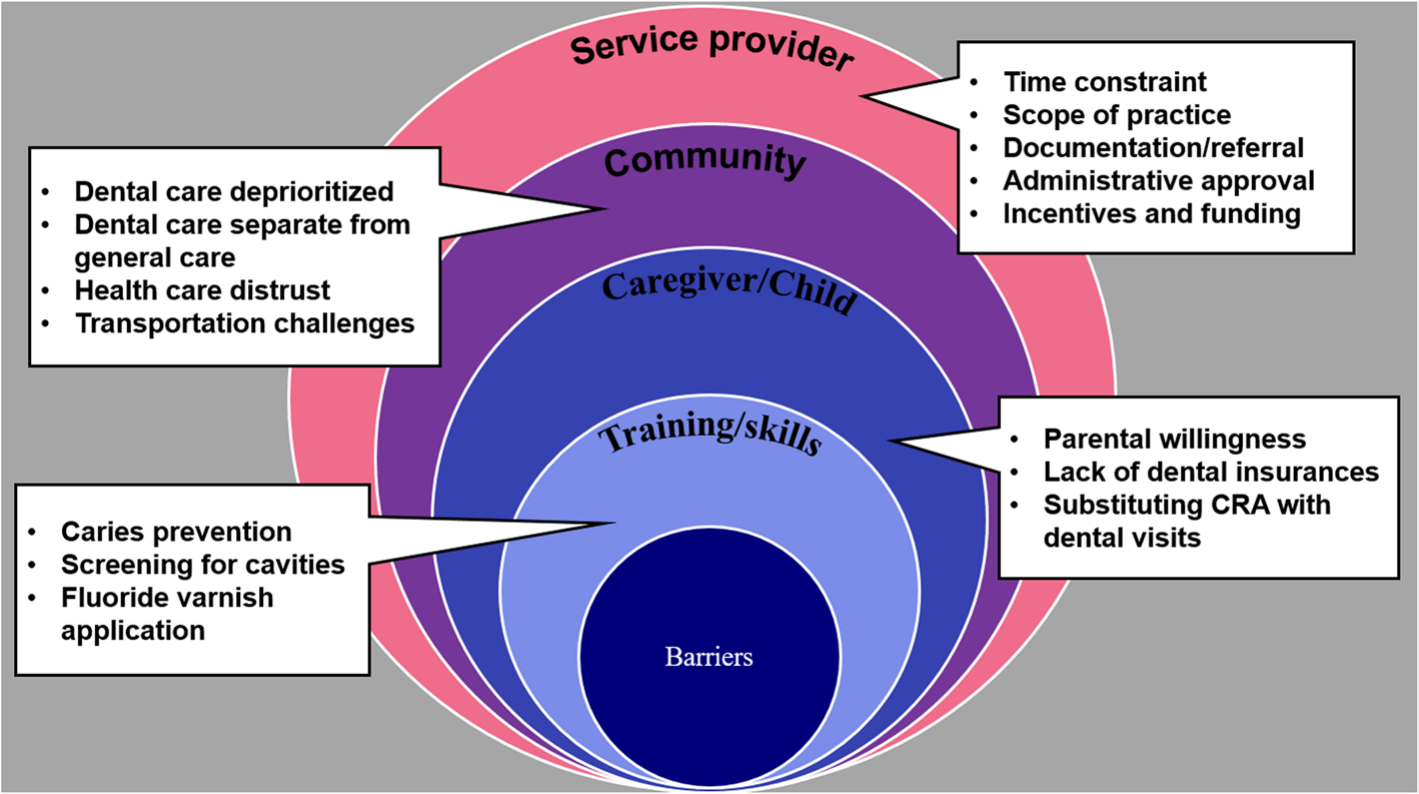
Themes and subthemes on barriers

Fifty NDPCPs participated in eight focus groups and twelve key-informant in-depth interviews. The participants consisted of 8 physicians, 31 public health nurses, 4 nurse practitioners, 2 physician assistants, 1 dietitian, and 4 child development workers. The mean age of the participants was 41 years (range: 24–61), while the mean years of practice was 13 years (range: 1–40). Thematic analysis of barriers to implementation and integration of the Canadian CRA tool into primary care revealed 4 major themes: service provider level, community level, caregiver and child level, and provider training and skills level barriers (Fig. 2). Quotes from interviews represent points raised by participants along with pseudonyms and professional designations (Table 1).

**Table 1:** Examples of quotes from participants

Themes	Sub-themes	Examples
Service provider level barriers	Time constraint	*“I would say as always, time is a constraint, time to get the product, to educate the client, to do the screening assessment that I think is an issue” (Edna, nurse)*
	Scope of practice	*“I think having support from the program to use it because I don't want to get in trouble and have people saying I'm working outside of my scope or I'm doing something that I shouldn't be doing” (Allice, nurse)*
	Documentation and referral pathways	*“I don't know of a formal process you have referring to specific dentists. Some of the nurse practitioners or doctors have referral forms that they would use, but other people outside of that aren't necessarily going to have a referral form other than a verbal like you need to go see the dentist here's where they are.” (Edna, nurse)*
	Administrative approval	*“I'm not sure about the varnish … how was that provided? Somebody has to pay for it, obviously. And how expensive is it and how do we go about getting it like those are just things I don't know” (Peter, Physician)*
	Incentives and funding	*“Unfortunately, in our program right now we're a little low on evaluation. There's a lot of things we do and our evaluation component is lacking and that's concerning” (Lola, nurse)*
Community level barriers	Dental care deprioritized	*““a lot of times the family or the client is directing the priority of care and sometimes, if they're fixated on specific issues that are high priority to them, it's very difficult to bring in something that they don't visualize as a priority and so it it's meeting the person where they're at and they might not be at this point.” (Nancy, nurse)” (Lola, nurse)*
	Separation of dental and general health	*“A lot of people don't think the doctor or the nurse are the people to bring up the dental caries, right” (Becky, nurse)*
	Lack of transportation	*“I think also transportation can be an issue for this so. I'm hoping it's something we can incorporate into our outreach and become more available” (Surat, nurse)*
	Healthcare distrust	*‘Some families may have not had great experiences with the healthcare system and maybe a little bit hesitant to take their children to get the help they need.” (Judy, nurse)*
Caregiver and child level barriers	Parental willingness	*“It's not going to be any problem applying the varnish. I mean I swabbed noses, which is probably worse than applying [fluoride] varnish but it also depends on the parent’s engagement, willingness.” (Edna, nurse)*
	Lack of dental insurance	*For the kids without treaty numbers, there's no [dental coverage], I think the biggest barrier is cost, it's not publicly funded. It's money out of parents’ pocket, most families are having to cough up some money and that is a barrier for some people (Sam, physician)*
	Substituting of CRA with actual dental visit	*“And also feel like this would be in place of going to see the dentist, right? Their perception of okay ‘I don't have to go to the Dentist because I got this right now” (Mary, nurse practitioner)*
Provider training/skills barriers	Caries prevention	*“Well, personally getting some additional training on recognition of pediatric dental caries, maybe some education on what the dentists do and what they offer and when they … What kind of interventions they do, and some more education on the consequences of childhood dental caries just for education purposes for the parents would be helpful” (Peter, physician)*
	Screening of tooth decay	*“We need a lot of education how to screen for that [caries] we also want…better access to professional screeners or to the appropriate dentist, or dental hygienist because we've run into barriers where we could screen but then the follow-up or to refer them to another professional could take a little longer. So that's where the barrier is” (Anna, nurse)*
	Fluoride varnish application	*“I would probably need more education on the actual process [fluoride vanish application] so that I could talk to the parents about why we would want to do it and the reasons behind so that I could promote it” (Precious, nurse)*

### Theme 1: service provider barriers

Service providers faced multiple challenges in implementing the CRA tool, both in their own workflows and in addressing parental barriers. While acknowledging the potential of the CRA tool to improve oral healthcare for Indigenous communities, participants cited time constraints, scope of practice concerns, documentation, and referral difficulties. Balancing caries risk assessment with other medical priorities was a common struggle. As one participant noted, *“Just time because we do have at the wellchild, other forms to fill out … if there's a time pressure, it would just look like challenge” (Betty, nurse).* Another added*, “If I'm seeing them coming in with cough, or different things … doing something like this would totally not be the main thing I need to deal with” (Mary, nurse practitioner).*

A key concern was the application of fluoride by non-dental providers. While some saw its benefits, others questioned whether it was within their role. One participant remarked*, “I don't know if that's our role [fluoride application] … is there a reason why whoever does it now can't continue doing that process?” (Sophia, nurse).*

Referral challenges also emerged, with poor communication from dental offices and a lack of clear referral pathways. *“That’s another thing … where, who are [we] referring them to, where are they going … I never really know” (Glory, nurse). Another added, “dentists don’t share information with us like other healthcare [providers] … we do not get those [reports] from dental offices” (Peter, physician).*

Limited funding was another major barrier, restricting access to fluoride application. One participant emphasized, *“If we had more funding … this is just something that could be easily made available to primary care clinics” (Josh, nurse practitioner).* Additionally, some providers felt unrecognized for the extra workload. As one explained*, “A lot of times we have add-ons to our work with no recognition” (Lola, nurse).*

### Theme 2: community level barriers

Participants identified several community-level barriers that may hinder the implementation and integration of the Canadian CRA tool in Indigenous pediatric primary care. These barriers include the perception of dental care as a low priority, the historical separation of dental and general healthcare, transportation challenges, and the impact of intergenerational trauma on healthcare trust and access.

A recurring challenge noted by participants was that dental care is often deprioritized in communities. Families struggling to meet basic needs may find it difficult to prioritize oral health, leading to lower engagement with dental care services. As one nurse described, *“Mine would be like what's the priority for the parents of these children, sometimes they're having a hard enough time just figuring out where they're going to get their next meal, is dentistry really a priority for them to go get their child’s teeth checked or finances to fund going to the dentist as well” (Fiona, nurse).* This highlights the reality that dental care often takes a backseat when families are dealing with food insecurity and financial strain.

Limited transportation options further exacerbate barriers to accessing dental care. Participants noted that logistical challenges, such as traveling with multiple children and the lack of reliable transportation, may create significant obstacles for families. This may hinder referrals to the dental provider for comprehensive dental treatment following initial CRA by NDPCPs. As one nurse explained, *“We also have to think like how do the family get there…the family may have 3–4 kids. How do you drag all these kids to the dental appointment? So that's some transportation…sometimes it seems like it's a simple thing, but it's a big barrier for some families” (Nancy, nurse).*

Another key barrier identified was the traditional separation of dental care from primary healthcare services. Participants noted that many parents do not perceive NDPCPs as suitable for delivering dental-related interventions. This systemic divide may create challenges in integrating the CRA tool into routine primary care visits. As one nurse practitioner highlighted, *“…a real challenge is that medicine and healthcare culturally and historically have been separate from our dentistry colleagues. So it can be difficult to integrate that into primary care because a lot of people like you know … [say] I care for this, we need the teeth … they go to the dentist, right?” (Josh, nurse practitioner).* This perception reinforces the idea that dental care is distinct from general health, complicating efforts to implement the CRA tool effectively.

Participants also pointed to the lingering effects of intergenerational trauma, which contribute to a deep-rooted distrust in the healthcare system. This mistrust affects health-seeking behaviors, including accessing dental care. Families who have experienced systemic discrimination and historical injustices may be reluctant to engage with healthcare providers, further limiting opportunities to provide preventive dental care.

### Theme 3: Caregiver and child-level barriers

Participants identified several barriers at the caregiver and child level that impact the implementation of the CRA tool in Indigenous pediatric primary care. These barriers, as perceived by the NDPCPs, include parental willingness to engage with the CRA tool during primary care visits, financial limitations due to lack of dental insurance, and misconceptions about the CRA tool serving as a substitute for actual dental visits.

One key barrier perceived by participants was parents' hesitancy to engage with the CRA tool due to concerns about being judged on their parenting practices. The CRA tool includes questions about feeding habits, such as the use of bottles at bedtime and the consumption of sugary snacks and drinks. Some caregivers may feel scrutinized when discussing these topics, making them reluctant to participate. As one nurse explained, *“Some people get defensive when you're talking about sugar intake or bottles or sippy cups so people tend to get pretty offended easily with those practices” (Edna, nurse).* Another participant highlighted the broader impact of this perception, stating, *“A lot of our families share that they feel very judged when they go to the dentist, especially if the caries was delayed or children are older or there's significant decay when they go. They feel very bad…and the care they receive that day can affect the care their child will receive for the rest of their life if they feel judged or not feel like they were treated respectfully or well” (Lola, nurse).* This underscores the need for a culturally sensitive and non-judgmental approach when discussing oral health with caregivers.

Participants also expressed concerns that financial constraints, particularly the lack of dental coverage, could prevent caregivers from following through on referrals prompted by the CRA tool. The tool is designed to identify children at risk for dental issues and refer them to a dental provider for comprehensive care if needed. However, without dental insurance or financial resources, families may not be able to access the recommended care. As one physician explained, *“I feel like I would be encountering a lot of problems in referral if they don't have insurance or they've got limited finances, I've got limited places that are going to accept payment plans so they don't really get to be autonomous in where they want to be referred to if their resources are limited because they don't have insurance” (Bayo, physician).* These financial barriers can limit access to follow-up care and reduce the effectiveness of the CRA tool in connecting children with necessary dental services.

Some participants raised concerns that parents might misunderstand the role of the CRA tool and preventive measures such as fluoride application, seeing them as substitutes for routine dental visits. This misconception could lead to reduced follow-through with dental referrals. One nurse highlighted this concern, stating, *“I would worry that if we did apply fluoride varnish, they [parents] would think they’re done and don’t need to go to the dentist, right? Because they’d say ‘you fixed it, I got the varnish so I’m good now and it’s not a priority anymore.’ I know that sounds silly, but that does happen” (Alice, nurse).* Addressing these misunderstandings is essential to ensuring that caregivers recognize fluoride varnish and CRA screening as complementary rather than replacement strategies for regular dental care.

### Theme 4: Provider training and skills barriers

Participants identified significant gaps in their knowledge regarding the causes, prevention, and screening for caries in children. Few had received formal training in early childhood oral health. A recurring concern was the need for education on fluoride application. As one participant explained, *“I think we could implement [Canadian CRA tool], but there's some things that need to happen first, right? We need to know about the [fluoride] varnish and we need to figure out that, otherwise, we would only be partially implementing” (Jane, nurse)*. Some participants expressed uncertainty about whether fluoride application was within their scope of practice but indicated a willingness to perform the procedure if authorized and adequately trained. One participant noted, “*I would feel if that was something that they wanted to put in our scope of practice, I would definitely want proper training” (Becky, nurse*). The desire for further education on fluoride use was expressed by all. Several participants suggested including guidance on aftercare instructions for parents regarding fluoride varnish. They also emphasized the need for training in child management techniques during screenings and fluoride application, particularly for young children. One participant reflected, *“but I think part of it too is even like, I don't know, are there specific techniques to help calm a child specifically while you're looking in their mouth, like behavioral techniques as well” (Surat, nurse)*.

## Discussion

This qualitative study provides valuable insights into the multifaceted barriers to implementing the Canadian CRA tool for preschoolers in Indigenous communities. These barriers span across service provider limitations, community-level challenges, caregiver engagement, and training needs. Addressing these issues is crucial for improving oral health outcomes and ensuring equitable access to care.

### Service provider barriers

Participants reported that integrating additional responsibilities such as fluoride varnish application into already busy primary care environments posed significant challenges. Key concerns included time constraints, unclear professional boundaries, documentation burdens, and limited funding. These findings align with prior studies that identified similar challenges, including staff shortages, inadequate infrastructure, and a lack of administrative support [13, 14, 19, 20]. Time constraints, in particular, are consistently noted as a major barrier to delivering oral health services in primary care [19].

Interestingly, studies outside the Indigenous context have shown promising outcomes for CRA integration. For example, a pilot study by the American Academy of Pediatrics (AAP) found that 80% of clinics reported the CRA tool was easy to use and required minimal workflow adjustments. Clinicians completed oral health assessments in under two minutes, and referrals for high-risk children increased significantly from 11 to 87% [21]. Similarly, a separate case study reported that oral health assessments added only 2–3 min to routine well-child visits, with time barriers successfully addressed through workflow optimization and staff training [22].

While these findings suggest CRA tools can be seamlessly incorporated into primary care, it is essential to consider the contextual differences. The AAP studies involved non-Indigenous clinics in the United States, where provider shortages, geographic isolation, and the need for culturally safe care may not be as prevalent. In contrast, Indigenous communities in Manitoba face unique systemic barriers, including limited access to dental services, historical mistrust in healthcare systems, and different healthcare priorities [23].

These contextual distinctions highlight the need for localized evaluation. It remains unclear whether the streamlined integration observed elsewhere would translate effectively to Indigenous primary care settings. Future studies measuring the time and effort required for NDPCPs to complete the Canadian CRA tool in Indigenous contexts would be particularly valuable. Such research could inform tailored workflow adaptations and implementation strategies.

Participants also identified documentation and referral challenges stemming from the lack of integrated electronic medical records (EMRs). Evidence suggests that embedding oral health tools into EMRs can enhance documentation, increase screening rates, and improve follow-up care for high-risk children [24]. Addressing provider concerns such as workload and compensation through policy initiatives like value-based payment models could further support oral care integration [25]. To mitigate provider barriers, strategies such as ensuring fluoride varnish availability, listing local dentists who accept publicly funded patients, creating clear CRA tool workflows, integrating the tool into EMRs, and appointing oral health champions within each clinic should be implemented [24, 26, 27].

### Community-level barriers

Participants highlighted that oral health is often a low priority for families facing socioeconomic challenges. This aligns with literature showing that food insecurity, housing instability, and poverty can reduce engagement with preventive dental care [28, 29]. Effective CRA implementation should be paired with broader supports such as food security programs and dental financial assistance to address families’ immediate needs alongside oral health education [30–32]. Transportation barriers were also cited, reflecting wider challenges in rural and Indigenous communities where distance and cost impede healthcare access [23, 33]. Community-driven solutions, such as mobile dental units, tele-dentistry, and transportation support programs, could help mitigate these barriers and facilitate follow-through on dental referrals [34].

The longstanding separation of dental and general healthcare also poses a significant challenge to CRA tool integration. Research shows that interprofessional collaboration, particularly co-location of dental and primary care services can lead to improved oral health outcomes [35]. Encouraging a paradigm shift where NDPCPs are seen as integral to early oral health intervention requires systemic efforts, including cross-disciplinary training, policy support, and community engagement to redefine expectations around primary care responsibilities in oral health.

Historical and ongoing experiences of colonization, including the legacy of residential schools and systemic discrimination, continue to shape Indigenous peoples’ relationships with healthcare. Participants emphasized the importance of culturally safe approaches, those that honor Indigenous values, knowledge, and autonomy as essential to rebuilding trust [36, 37]. Strategies like involving Indigenous health workers, supporting community-led oral health initiatives, and promoting trauma-informed care are vital to improving CRA adoption and healthcare equity [3, 37].

### Caregiver and child-level barriers

Beyond community-level barriers, caregiver and child-specific factors also play a crucial role in the implementation of the CRA tool. Participants expressed concerns that some caregivers may be hesitant to engage with the CRA tool due to fears of judgment related to parenting practices. Questions related to feeding routines, bottle use, and sugary snacks can feel intrusive or stigmatizing, leading to reluctance of caregivers to engage. Prior research supports that perceived judgment from healthcare providers can lead to defensiveness and decreased engagement [34, 36, 38, 39]. A respectful, culturally sensitive communication style is essential to building trust and promoting participation.

Financial constraints further limit caregivers’ ability to follow through with dental referrals generated by the CRA tool. Many Indigenous families face challenges due to a lack of dental insurance or limited financial resources. Participants highlighted difficulties in referring patients when only a few dental providers accept publicly funded coverage or offer flexible payment plans. These financial limitations are consistent with existing literature, which notes that economic barriers significantly hinder dental care access in Indigenous communities [5, 23]. Strengthening and expanding public programs such as the interim Canada Dental Benefit and the Canadian Dental Care Plan alongside targeted outreach to connect families with available resources, could significantly improve referral follow-through [32, 40].

Another critical concern was the misconception that the CRA tool and preventive measures, such as fluoride application, could replace routine dental visits. Some caregivers may assume that once fluoride varnish is applied, no further action is necessary. Literature on oral health interventions suggests that effective patient education is key to preventing such misunderstandings [41]. Providers should emphasize that CRA screening and fluoride application serve as complementary strategies rather than replacements for professional dental care, reinforcing the importance of routine checkups.

### Provider training and skills barriers

Participants frequently cited limited training in caries screening and fluoride application as a barrier to effective implementation. This is consistent with literature indicating that non-dental providers often receive minimal instruction in pediatric oral health despite their frontline role in early intervention [19].

Nonetheless, many providers demonstrated a commitment to oral health by offering parent education, even without formal training. This speaks to the potential of NDPCPs to play a meaningful role in early childhood oral care [11, 42]. Strengthening this potential requires integrating oral health into medical and nursing education, supported by refresher training during onboarding. Hands-on training was particularly emphasized, especially for fluoride application and child behavior management techniques. Such training has been shown to increase provider confidence and uptake of preventive services [43]. Uncertainty about scope of practice emerged as a key barrier, with providers willing to apply fluoride if authorized. Clear policies and guidelines are needed to support role expansion and clarify responsibilities.

Strategic leadership is crucial for CRA integration [22]. Engaging champions—such as public health nurses, dental hygienists, and clinic managers—can drive adoption through staff training, ongoing education, and workflow integration [24, 26, 44]. Structured support, clear policies, and leadership engagement will enhance provider confidence and promote sustainable implementation. Prioritizing these elements will facilitate CRA tool adoption in primary care, improving early childhood oral health outcomes.

### Strengths and limitations

A key strength of this study is its qualitative design, which allowed for rich, in-depth exploration of healthcare providers' perspectives across multiple levels. These insights offer a comprehensive understanding of both the practical and systemic challenges to CRA implementation. The inclusion of diverse provider roles from different care settings adds to the depth of the findings. However, the study has limitations. The small sample size may restrict generalizability, and Indigenous communities in Manitoba may not fully represent broader Indigenous populations. Participant responses may have been influenced by social desirability bias, and no triangulation with secondary data sources such as policy documents was conducted. Future studies may consider incorporating such data to reinforce the findings. Additionally, the study focused on provider perspectives and may not fully capture the experiences of Indigenous families and communities. Regional differences in healthcare delivery systems may also affect applicability.

## Conclusion

The successful implementation of the Canadian CRA tool in Indigenous communities requires a holistic, context-sensitive approach that addresses provider, community, caregiver, and systemic barriers. Collaborative leadership, targeted training, culturally safe care, and integration of oral health into broader support systems are key to improving uptake and outcomes. By prioritizing equity and honoring Indigenous perspectives, these efforts can contribute meaningfully to reducing health disparities. Future research and pilot initiatives are needed to assess feasibility, inform best practices, and guide broader implementation.

## Supplementary Information

Supplementary Material 1.

## Data Availability

The datasets generated and analyzed during the current study are available from the corresponding author on reasonable request. Data is available at Children's Hospital Research Institute of Manitoba, University of Manitoba shared drive and will be shared on reasonable request.
